# Reevaluating the total genotype score: a practical perspective from applied sport science

**DOI:** 10.1007/s13353-025-01020-z

**Published:** 2025-10-01

**Authors:** Ricardo Muller Bottura, Daniel Blasioli Dentillo

**Affiliations:** 1 Department of Neurosciences, Biomedicine and Movement Sciences, Università Degli Studi Di Verona, 37124 Verona, Italy; 2 Department of Neuroscience, Rehabilitation, Ophthalmology, Genetics, and Maternal-Infant Sciences (DINOGMI), Università Degli Studi Di Genova, 16132 Genoa, Italy; 3 DGLab, Ribeirão Preto, 14056-680 Brazil

**Keywords:** Total Genotype Score, Genetic Profile, High-Performance, Athletes, Running

## Abstract

Over the last few years, the number of publications regarding genetics and sports has notably increased. However, the way this information is actually used is often misinterpreted by those who do not apply genetics in their day-to-day training. Therefore, the purpose of this short communication is to exemplify, using the real cases of identical twin athletes, how genetic information must be applied in sports. Additionally, we present data on performance tests and individual preferences for training and competitions between the athletes. Accordingly, the study concludes with a distinction between genetic profile studies and genetic application studies, emphasizing the importance of further research in this area, especially case reports.

## Introduction

There has been a notable increase in scientific publications involving genetics and sports over the last few years, as evidenced by at least three systematic reviews demonstrating the relationship between several polymorphisms and athletic performance (Bray et al. 2009 Jan; Ahmetov et al. 2016; Semenova et al. 2023). Despite this body of research, the gap between scientific findings and their practical applications remains significant, partly because ongoing studies continue to focus on genetic profiling methods, such as the Total Genotype Score (TGS), which aggregates multiple genetic markers to identify athletic talent (Pranckeviciene et al. 2021; Homma et al. 2022). However, there are valid reasons to be careful about relying too much on genetic data to predict athletic ability and performance (Vlahovich et al. 2017; Varillas-Delgado et al. 2022).

In this short communication, we present an alternative approach to applying genetic information in sports training, illustrated by the case of two 18-year-old female identical twin athletes. These athletes began training at age 8, consistently following the same training schedules and participating in the same competitions over the past decade. Despite sharing an identical genome and similar training histories, they developed distinct preferences in training volume, intensity, and the competitive distances they favor. Using this case study as a framework, the aim of this short communication is to demonstrate: (a) why strategies that focus on genetic profiles, such as the TGS, should not be used in sports; (b) how to effectively apply genetic information in sports training; and (c) the importance of case reports in enhancing our understanding of its application.

## Methods

### Participants

The case involves two 18-year-old female identical twin athletes who have trained in track and field since the age of 8. Our professional involvement with them occurred between 2022 and 2024, during their final youth phase (ages 16 to 18). During this period, both athletes consistently followed the same training schedules under the same coaching staff and participated in the same competitions. Their anthropometric measurements and physiological responses were periodically monitored, providing a comparable dataset to explore practical applications of genomic information in sports (Table 1). Although the athletes are referred to as identical twins based on family history and strong physical resemblance, no molecular zygosity test was conducted. We considered such testing unnecessary given the study’s scope, which does not aim to draw conclusions based on zygosity, but rather to examine the limitations of the TGS model in practical settings.

**Table 1:** Anthropometric Measurements and Incremental Test Results of the Athletes

	September 2022		May 2023	
	Athlete 1	Athlete 2	Athlete 1	Athlete 2
Weight (kg)	41.4	47.5	39.9	46.4
Height (m)	1.55	1.60	1.55	1.59
Maximum Velocity (km/h)	18.0	18.0	18.0	19.0
Maximum Heart Rate (bpm)	186	185	175	180
VO_2_ max (ml/kg/min)	54.6	52.1	57.7	57.6

The running protocol began at 9 km/h, with a 1 km/h increment every minute

### Genetic analysis

Buccal cell samples were obtained using oral swabs and analyzed in a certified laboratory via real-time polymerase chain reaction (qPCR) using the QuantStudio 6 platform (Thermo Fisher Scientific). Customized primers and probes were employed to genotype 20 specific polymorphisms selected based on their known association with performance-related traits (Ahmetov et al. 2016; Semenova et al. 2023). All gene variants, genotype interpretations, and scoring criteria are detailed in Table 2.

**Table 2:** Genes map and Total Genotype Score of Athletes

Genes	Genotypes	Genotype scores	Athlete’s genotypes
ACTN3 (rs1815739)	RR and RX—production of alpha-actinin-3	XX = 0; RX = 1; RR = 2	RX = 1
	XX—absence of alpha-actinin-3		
MCT1 (1,049,434)	A/A—higher production of MCT1	TT and TA = 0; AA = 2	TA = 0
	T/T and A/T—lower production of MCT1		
AGT (rs699)	C/C and T/C—higher production of angiotensinogen	CC = 0; TC = 1; TT = 2	CC = 0
	T/T—lower production of angiotensinogen		
ACE (rs4341)	G/G and C/G—higher production and action of ACE	GG = 0; CG = 1; CC = 2	CC = 2
	C/C—lower production and action of ACE		
BDKRB2 (rs1799722)	T/T—higher quantity of bradykinin receptor	CC = 0; TC = 1; TT = 2	TC = 1
	C/T and C/C—lower quantity of bradykinin receptor		
NOS3 (rs2070744)	T/T and C/T—higher production of NOS3 enzyme	CC and TC = 0; TT = 2	TC = 0
	C/C—lower production of NOS3 enzyme		
NOS3 (rs1799983)	G/G—higher activity of NOS3 enzyme	TT = 0; GT = 1; GG = 2	GT = 1
	T/T and G/T—lower activity of NOS3 enzyme		
ADRB2 (1,042,713)	A/A—higher affinity of receptor to adrenaline	GG = 0; AG = 1; AA = 2	GG = 0
	A/G and G/G—lower affinity of receptor to adrenaline		
PPARA (rs4253778)	G/G and G/C—higher energy production from lipid metabolism	CC = 0; GC = 1; GG = 2	GG = 2
	C/C—lower energy production from lipid metabolism		
PPARD (rs20165209)	T/T and C/T—stimulates more PPARGC1A expression	CC = 0; CT = 1; TT = 2	CT = 1
	C/C—stimulates less PPARGC1A expression		
PPARD (rs2267668)	A/A and A/G—stimulates more PPARGC1A expression	GG = 0; AG = 1; AA = 2	AG = 1
	G/G—stimulates less PPARGC1A expression		
PPARGC1A (rs8192678)	C/C and C/T—higher production of mitochondria	TT and CT = 0; CC = 2	CT = 0
	T/T—lower production of mitochondria		
VEGFA (rs2010963)	C/C and C/G—higher production of VEGF-A	GG = 0; CG = 1; CC = 2	CG = 1
	G/G—lower production of VEGF-A		
TNFA (rs1800629)	G/G—lower production of TNF-A	GG = 0; AG = 1; AA = 2	GG = 0
	A/A and A/G—higher production of TNF-A		
IL6 (rs1800795)	C/C—lower synthesis of IL-6	CC = 0; CG = 1; GG = 2	CG = 1
	G/G and C/G—higher synthesis of IL-6		
CRP (rs1205)	T/T—lower production of CRP	CC = 0; CT = 1; TT = 2	TT = 2
	C/C and C/T—higher production of CRP		
SOD2 (rs4880)	C/C and T/C—higher activity of SOD2 enzyme	TT = 0; TC = 1; CC = 2	CC = 2
	T/T—lower activity of SOD2 enzyme		
CAT (rs1001179)	C/C—higher activity of CAT enzyme	TT = 0; TC = 1; CC = 2	CC = 2
	T/T and C/T—lower activity of CAT enzyme		
GPX1 (rs1050450)	C/C—higher activity of GPX1 enzyme	TT = 0; TC = 1; CC = 2	CC = 2
	T/T and C/T—lower activity of GPX1 enzyme		
COL5A1 (rs12722)	C/C—produces COL5A1 with adequate structure	TT = 0; TC = 1; CC = 2	TC = 1
	C/T and T/T—produces COL5A1 with altered structure		

Presentation of all genes, interpretation of each genotype according with literature. TGS score (50) was calculated using the formula from Williams and Folland (2008)

The TGS was calculated using the formula:

$$TGS= \frac{100}{number\; of\; alleles} \times\Sigma (genotypes)$$

This method was based on the model proposed by Williams and Folland (2008), incorporating relevant polymorphisms reported in the literature.

### Preference questionnaire

Individual training and competition preferences were assessed using a custom questionnaire developed by the authors to reflect practical aspects of athlete behavior observed in high-performance settings. Although not formally validated, this tool was used retrospectively to document real-world training tendencies that emerged over the athletes’ careers. The full set of questionnaire items and the athletes’ responses are presented in Results.

### Ethical considerations

This case report was based on retrospective data collected during the authors’ professional follow-up with the athletes between 2022 and 2024. No procedures were conducted for research purposes, and no interventions were performed beyond routine training or assessments. Written informed consent was obtained from both athletes and their legal guardians at the time of genetic material collection, including consent for the use of anonymized data in scientific publications. All personal data were anonymized prior to analysis. According to Brazilian Resolution CNS 510/2016 (Article 1, Section I), research using secondary or anonymized data collected for non-research purposes is exempt from mandatory ethics committee review, provided that ethical principles are respected. This study also complies with the Declaration of Helsinki and the Brazilian General Data Protection Law (LGPD – Law No. 13.709/2018).

### Physiological assessment

The physiological data presented in Table 1 refers to the maximal values reached during maximal incremental treadmill test. The incremental test began at either 9 or 10 km/h depending on the testing session, with speed increased by 1 km/h every minute (or 0.5 km/h in the early stages in one session), and was performed until volitional exhaustion. The tests were performed under controlled laboratory conditions (temperature: 21–23 °C; humidity: ~ 50%), on a treadmill with 1% incline. Heart rate was continuously monitored (Polar H10), and rating of perceived exertion was recorded using the OMNI Walk/Run scale.

## Results

### Performance data

Performance metrics, including national-level achievements, are provided in Table 3. Despite sharing an identical TGS of 50, both athletes achieved notable performance results and were selected for national youth teams.

**Table 3:** Results of Competitions in Which Both Athletes Competed Together from 2022 to 2024

Distance	2022		2023		2024	
	Athlete 1	Athlete 2	Athlete 1	Athlete 2	Athlete 1	Athlete 2
1500	2nd place	-	-	3 × 1 st place	-	1 st place
3000	3rd place	5th place	2nd place	1 st place	-	1 st place
5000	-	-	1 st place	-	3rd place	-
6000	2nd place	3rd place	-	-	-	-

The results presented in this table pertain exclusively to national-level competitions

### Training preferences

Details regarding training and competition preferences are presented in Table 4. Despite identical genetic makeup and training exposure, the athletes demonstrated distinct individual preferences, further supporting the notion of phenotypic divergence due to environmental and psychological factors.

**Table 4:** Athletes’ preferences of training and distances

Questions	Athlete 1 responses	Athlete 2 responses
What is your favorite distance?	3000 m and 5000 m	1500 m
What type of training do you prefer: long or short?	Long with high intensity	Short with high intensity
What distances do you compete in currently?	1500 m, 3000 m, and 5000 m	800 m, 1500 m, 3000 m, and 5000 m
What type of training do you believe you dedicate yourself to the most?	Stronger intervals (400 m, 1000 m, and 2000 m)	5 × 400 m
Do you enjoy strength training?	I don't like it	I don't like it
If you were to practice another sport, what would it be?	Triathlon	None
What distance would you like to focus on for the rest of your career?	5000 m	1500 m

This questionnaire was completed by the athletes individually, without the presence of the other, and without time to communicate regarding their responses

## Discussion

### Genetic profile studies

In 2008, the concept of a genetic profile linked to endurance performance was introduced to the scientific community (Williams and Folland 2008). In this paper, the authors developed what they termed the "Total Genotype Score" (TGS), based on alleles from 23 genes and their reported associations with endurance performance in the literature. While this initially seemed like a promising idea, it oversimplified a much more complex reality. By assigning equal weight to each gene, the TGS does not adequately consider the physiological roles of the proteins produced during training adaptations or the genetic differences among populations. This limitation creates a misleading perception that it is possible to predict athletic success or establish a definitive relationship between an ideal genetic profile and a specific sport (Yvert et al. 2016).

A cross-sectional study of Brazilian athletes found an increased odds ratio of having a TGS ≥ 60 and being an elite athlete compared to those with a score ≤ 55.0 (Guilherme and Lancha 2020). However, only 32.6% of elite athletes reached this TGS level (which ranges from 20 to 90). Using the formula proposed by Williams and Folland (2008), we calculated the TGS for the twin athletes, which resulted in 50 points. Although this score falls within the average range for Brazilian elite athletes, it does not appear to be associated with their athletic achievements. Both athletes reached national-level performance and represented the Brazilian youth team, suggesting that TGS alone is not predictive of elite success.

Another common use of the TGS in scientific literature is the attempt to create an ideal genetic profile for specific sports (Ruiz et al. 2009). While this might offer some insights, the variability in population genetics limits the applicability of a universal model, as shown by studies failing to replicate results across ethnic groups (Yvert et al. 2016; Miyamoto-Mikami et al. 2017).

### Genetic application studies

Several studies have examined how specific polymorphisms influence athletic performance and physiological responses to training (Kikuchi and Nakazato 2015; Eynon et al. 2013; Thomas et al. 2012). Genes such as ACTN3 are widely recognized for their influence on muscle function and fiber type composition (Coso et al. 2017; Pickering and Kiely 2017; Appel et al. 2021). In the context of our case, both athletes shared identical genotypes, but expressed distinct training preferences, something the TGS could not predict. This outcome is not unexpected, given that sports performance is a complex phenotype influenced by multiple genetic and non-genetic factors. However, we emphasize this result precisely because a considerable portion of the current literature and commercial genetic testing still relies on oversimplified models such as the TGS to make individual predictions. Our findings highlight the risks of this reductionist approach and support the need for more nuanced and integrative methods when applying genetics in sport.

An applied approach involves categorizing genes by physiological function, aligning training prescriptions to enhance favorable responses or mitigate genetic limitations (Ginevičienė et al. 2022; Puthucheary et al. 2011). For example, variations in ACE, NOS3, and BDKRB2 genes affect vasodilation and oxygen delivery (Fuentes et al. 2002; Rigat et al. 1990; Yusof et al. 2018; Wong 2016; Murphey et al. 2000; Ignjacev-Lazich et al. 2005; Saunders et al. 2006; Williams et al. 2004), while MCT1 and ACTN3 influence lactate transport and muscle contraction efficiency (Dubouchaud et al. 2000; Fedotovskaya et al. 2014; North and Beggs 1996; North et al. 1999). This framework promotes individualized training while respecting the multifactorial nature of adaptation.

These findings underscore the inadequacy of using the TGS as a standalone tool. Instead, integrating genomic data into a broader context, alongside field tests, subjective preferences, and coach-athlete communication, can enhance training personalization.

### Importance of case reports

While case reports do not support generalization, they offer unique insights into complex, real-world situations. Randomized controlled trials in elite athletes are difficult due to logistical and ethical constraints (Halperin 2018). Presenting two genetically identical athletes with divergent preferences and outcomes illustrates the dynamic interplay between genotype and environment.

Other twin-based studies have also explored this gene–environment interaction. Klissouras et al. (2001), in a co-twin study involving elite-level athletes, found that although genetic background strongly influenced certain physiological capacities, final athletic performance was determined by a multifactorial combination of training, motivation, environment, and opportunity. This supports the notion that genetic information explains only part of the performance phenotype, and that efforts to reduce it to a single predictive score (such as the TGS) oversimplify its role.

Case studies like this help bridge the gap between molecular findings and applied practice, highlighting the limitations of reductionist models and the value of individualized approaches in performance science (Halperin 2018; Georgiades et al. 2017).

## Conclusion

This case study involving genetically identical twin athletes challenges the use of TGS as predictive tools for sport selection or talent identification. Despite sharing the same genetic profile, the athletes developed different preferences, strengths, and competitive focuses. This observation underscores the importance of gene-environment interactions and the limitations of deterministic interpretations of genetic data. While genomic insights may contribute to individualized training strategies, they should never be used in isolation. Importantly, gene-environment interactions will always play a central role in shaping athletic performance, and acknowledging their inevitability is essential to avoid deterministic or reductionist interpretations. Our findings, therefore, support a cautious, integrative approach to applying genetic information in sport science, one that respects the multifactorial nature of human performance and avoids oversimplified models.

## Data Availability

The datasets generated and/or analyzed during the current study are available from the corresponding author on reasonable request. Any additional information related to this study that supports the findings reported is available from the authors upon request, ensuring compliance with privacy and ethical restrictions.
